# LncRNA Bmp1 promotes the healing of intestinal mucosal lesions via the miR-128-3p/PHF6/PI3K/AKT pathway

**DOI:** 10.1038/s41419-021-03879-2

**Published:** 2021-06-09

**Authors:** Mengmeng Zhuang, Yuequ Deng, Wenwen Zhang, Bo Zhu, Hao Yan, Jiaqi Lou, Pan Zhang, Qingwei Cui, Hao Tang, Han Sun, Yong Sun

**Affiliations:** 1 Department of Burn Surgery, the Affiliated Huaihai Hospital of Xuzhou Medical University, Xuzhou, 221004 Jiangsu Province China; 2 Department of Burn Surgery, the 71st Group Army Hospital of PLA, Xuzhou, Jiangsu Province China

**Keywords:** Cell growth, Cell migration

## Abstract

Intestinal mucosal injuries are directly or indirectly related to many common acute and chronic diseases. Long non-coding RNAs (lncRNAs) are expressed in many diseases, including intestinal mucosal injury. However, the relationship between lncRNAs and intestinal mucosal injury has not been determined. Here, we investigated the functions and mechanisms of action of lncRNA Bmp1 on damaged intestinal mucosa. We found that Bmp1 was increased in damaged intestinal mucosal tissue and Bmp1 overexpression was able to alleviate intestinal mucosal injury. Bmp1 overexpression was found to influence cell proliferation, colony formation, and migration in IEC-6 or HIEC-6 cells. Moreover, miR-128-3p was downregulated after Bmp1 overexpression, and upregulation of miR-128-3p reversed the effects of Bmp1 overexpression in IEC-6 cells. *Phf6* was observed to be a target of miR-128-3p. Furthermore, PHF6 overexpression affected IEC-6 cells by activating PI3K/AKT signaling which was mediated by the miR-128-3p/PHF6 axis. In conclusion, Bmp1 was found to promote the expression of PHF6 through the sponge miR-128-3p, activating the PI3K/AKT signaling pathway to promote cell migration and proliferation.

## Introduction

The intestinal mucosa functions physiologically as a defensive barrier, preventing bacteria and endotoxins (usually present in the gastrointestinal tract) from accessing extraintestinal tissues and organs. Maintaining the normal intestinal barrier function requires complex interactions of various defense mechanisms, including the ecological balance of the local intestinal flora, peristalsis, preservation of the mucus layer and epithelial cell barrier, and maintenance of normal epithelial cell turnover, immune function, and the enterohepatic axis. In addition, the loss of the intestinal barrier function appears to play a role in the development of systemic infections and/or multiple organ failure (MOF) in some patients^1^. Therapeutic measures that help support the intestinal barrier function include maintaining an effective circulating blood volume, rapid identification and control of infection, the appropriate use of antibiotics, and optimal nutritional support. Despite the variety of strategies to restore the intestinal barrier function, effective treatment is currently limited. Therefore, elucidating the key molecular mechanisms mediating intestinal mucosal injury will facilitate treatment and improve prognosis.

RNA molecules that are not translated into proteins are called non-coding transcripts. These long non-coding RNAs (lncRNAs) represent a class of transcripts that are longer than 200 nucleotides and have no protein-coding capabilities^2,3^. Recently, many lncRNAs related to the intestinal mucosa have been identified by high-throughput analysis of expression profiles. For example, downregulation of lncRNA ANRIL in the intestinal mucosa is associated with tumor risk, tumor activity, and increased levels of proinflammatory cytokines^4^. lncRNA H19 can inhibit the expression of p53 protein, microRNA 34a, and let-7, and promote the proliferation and epithelial regeneration of intestinal epithelial cells (IECs)^5^. In our previous study, we found, using lncRNA microarray, that lncRNA Bmp1 expression was significantly increased in the intestines of burned mice. The length of mouse Bmp1 is 4464 bp and its gene is located on chromosome 14qD2 (https://www.genome.ucsc.edu/). It was first discovered in bone and reported to play multiple functions in bone formation. At present, there are few studies on Bmp1, and it is not clear whether Bmp1 can promote the repair of intestinal mucosa in burned mice.

Recent studies have shown that lncRNA can interact with miRNA to become a competitive endogenous RNA (ceRNA), thereby regulating the expression of target genes^6^. MicroRNAs (miRNAs) play important roles in gene regulation by binding to the 3′-untranslated region (3′UTR) of the target mRNA, which in turn leads to mRNA degradation or inhibition of translation^7^. For example, lncRNA-1604 inhibits miR-200c, resulting in Zinc finger E-box binding homeobox (ZEB) overexpression and promoting embryonic stem cell differentiation^8^. LncRNA H19 regulates the expression of its target spaghetti via sponge miR-141^9^. MiR-128-3p is a brain-rich microRNA that is highly enriched and upregulated in the embryonic mouse brain. It is thought to be involved in the control of neurogenesis and synaptogenesis. It has been reported that miR-128 regulates neuronal migration and growth through the gene *Phf6*^10^. We found in the pre-experiment that Bmp1 contained two binding sites of miR-128-3p. However, it is not clear whether Bmp1 can promote proliferation and migration through sponge adsorption miR-128-3p.

Plant homeodomain finger protein 6 (PHF6), located on Xq26-q27, was initially reported as a single gene mutation in Börjeson–Forssman–Lehmann syndrome, which is characterized by X-chromosome-related intellectual disabilities^11^. PHF6 encodes a plant homology domain (PHD) factor containing four nuclear localization signals and two incomplete PHD zinc finger domains^12^. Studies have shown that PHF6 may significantly promote proliferation and migration and the epithelial-mesenchymal transition (EMT) in hepatocellular carcinoma (HCC) cells^13^. PHF6 might play a crucial role in mouse neuronal migration during the early phases of development in primary cortical neurons and the developing mouse brain^14^. It is reported that the 3′UTR of the *Phf6* gene contains a sequence complementary to the seed region of miR-128-3p, suggesting that miR-128-3p can directly target *Phf6*^10^. Therefore, we speculate that miR-128-3p may play a physiological role through PHF6.

The PI3K/AKT pathway plays a role in a variety of physiological activities, and its activation is closely related to cell proliferation, survival, migration, invasion, metastasis, and tumor growth^15^. The activation of the PI3K/Akt pathway can enhance the wound healing response, increase the expression of EMT markers (MMP 9), and decrease the expression of cell adhesion/epithelial markers (ZO-1, E-cadherin)^16^. Matrix metalloproteinases (MMPs) can degrade all components of the extracellular matrix (ECM) and play key roles in normal physiological processes involving ECM remodeling, such as wound healing, angiogenesis, and development. MMP-9 has been shown to be involved in the migration of several cell types, namely macrophages, T lymphocytes, and eosinophils, through the recombinant basement membrane^17^. Zonula occludens (ZO)−1 is a multi-domain scaffold protein, which is known to play a key role in the targeting, anchoring, and aggregation of cross-linked cell adhesion molecules and cytoskeleton proteins, as well as in the establishment of cell–cell adhesion and the maintenance of stable tissue structure^18^. E-cadherin plays an important role in cell–cell adhesion, epithelial-to-mesenchymal transformation, cell migration, and invasion^19^.

In this study, we explore the effects of Bmp1 on cell proliferation and migration by inducing overexpression of Bmp1. We have also investigated the associated mechanism to determine whether Bmp1 works through a competing endogenous RNA (ceRNA) mechanism to reduce miR-128-3p bioavailability and induce the transcription of the miR-128-3p target gene *Phf6*, which significantly increases the phosphorylation levels of PI3K and AKT. Our results provide a reference for further analysis of the lncRNA-miRNA-mRNA-signaling pathway network during intestinal mucosal injury repair.

## Results

### Bmp1 expression increases in the burned mouse model

**Fig. 1:**
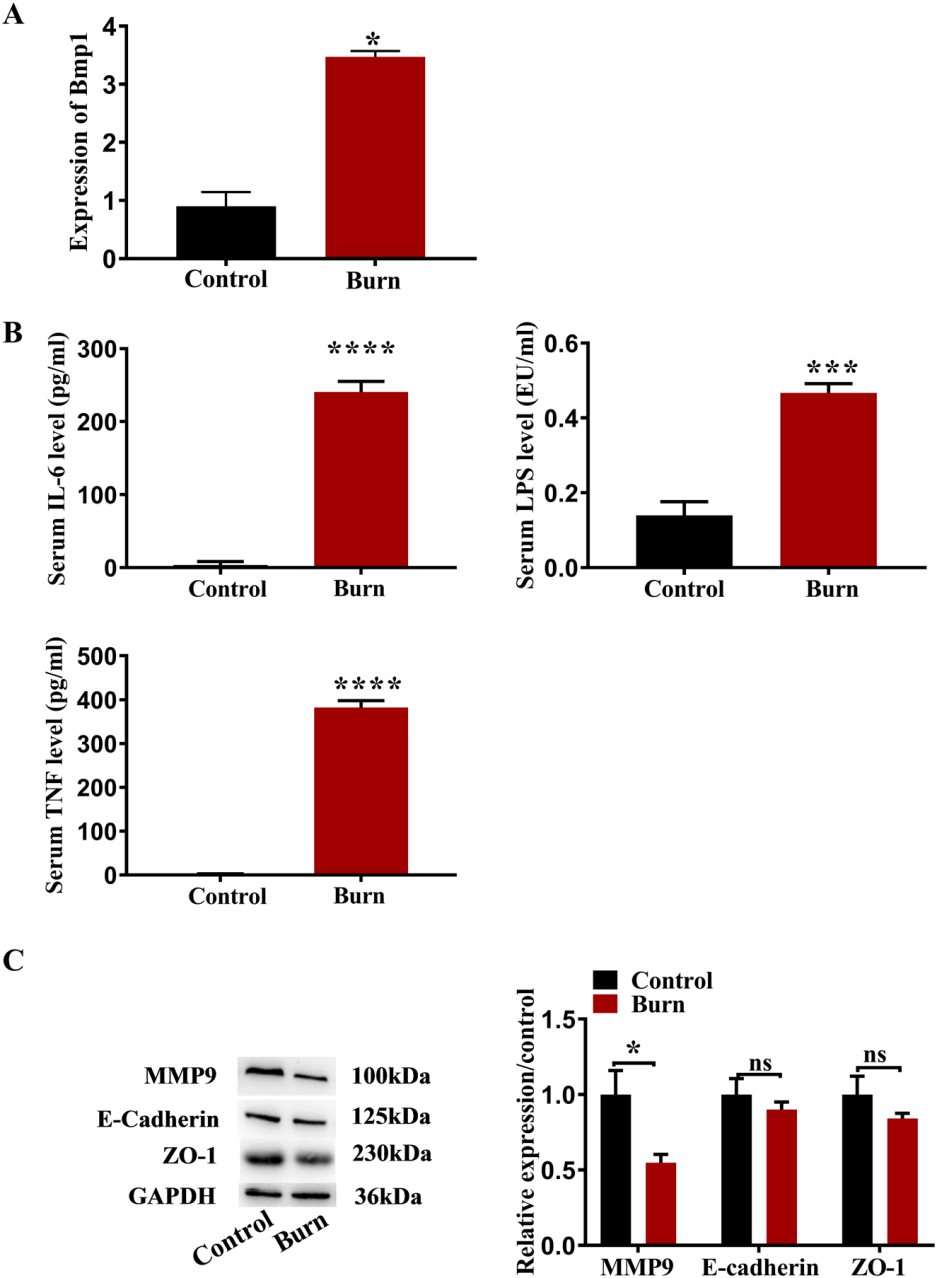
Expression of Bmp 1, TNF-α, IL-6, LPS, MMP-9, E- cadherin, and ZO-1 in intestinal tissue of burned mice. **A** Expression of Bmp1 (*n* = 10), **B** TNF-α, IL-6, LPS (*n* = 10), **C** cell migration-related protein MMP9, E-cadherin, and ZO-1 were detected by qRT-PCR, ELISA, and western blot, respectively (*n* = 10). All data were demonstrated as mean ± standard deviation (SD) from three independent experiments performed in duplicate. *p < .05, ***p < .001, and ****p < .0001 were all significant differences.

To detect changes in Bmp1 expression, intestinal mucosal RNA was extracted from burned mice. qRT-PCR results showed that the expression level of Bmp1 in the intestine of mice 24 h after burn was significantly higher than that in the control group (Fig. 1A). ELISA results showed that the levels of serum LPS, IL-6, and TNF- α in the burn group were significantly higher than those in the control group (Fig. 1B). Western blot results showed that the expression level of the migration-related protein MMP9 in the intestines of mice 24 h after burn was significantly lower than that in the control group, while the expression levels of E-cadherin, and ZO-1 were slightly lower than those in the control group (Fig. 1C).

### Bmp1 upregulation promotes cell proliferation and migration

**Fig. 2:**
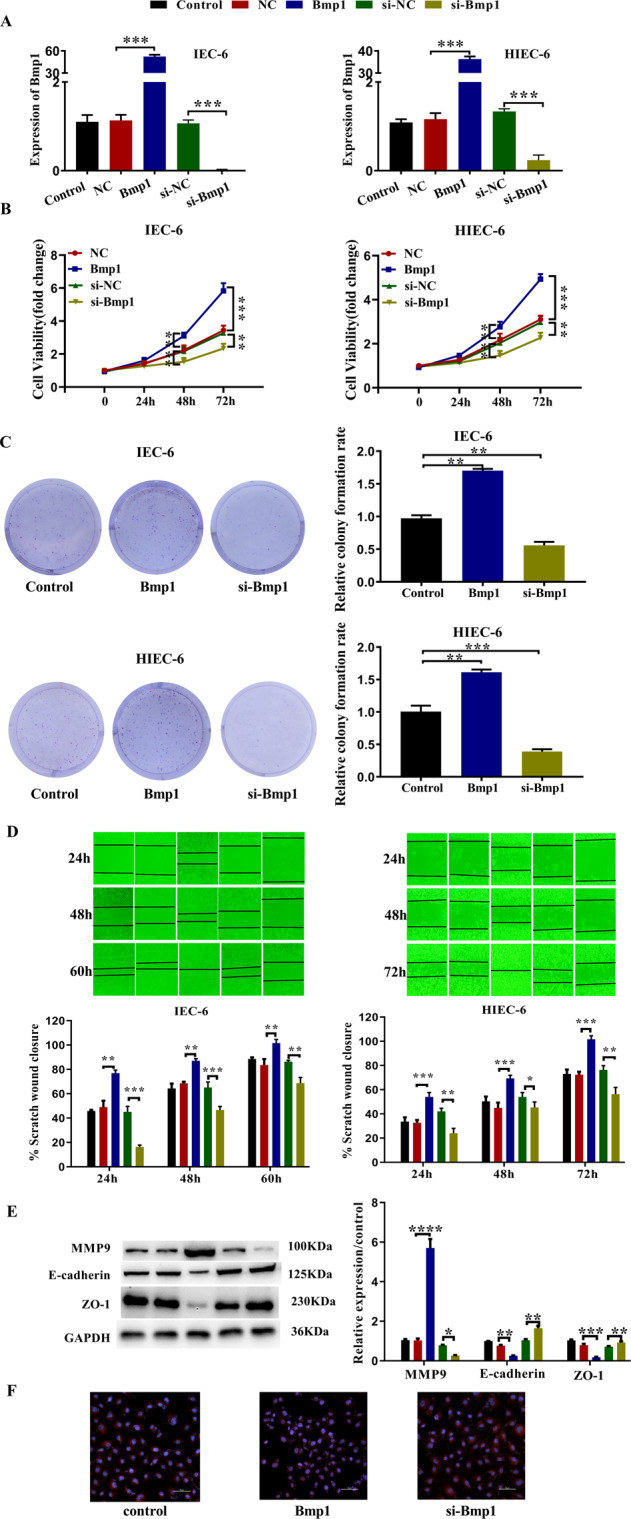
Overexpression of Bmp1 promotes the migration and proliferation of IEC-6 cells. **A** The alter expression of Bmp1 was determined using qRT-PCR (*n* = 3). **B** Cell viability (*n* = 3), **C** colony formation assays were performed to exam the function of Bmp1 in cells proliferation. **D** Cell migration ability was quantified by the percentage of the area of cells migrated to the wound in the total wound area (*n* = 3). **E** MMP9, E-cadherin, and ZO-1 were detected by western blot (*n* = 3). **F** Representative immunofluorescence analysis showed the content of E-cadherin in treated IEC-6 cells (*n* = 3). All data were demonstrated as mean ± SD from three independent experiments performed in duplicate. *p < 0.05, **p < 0.01, ***p < 0.001, and ****p < 0.0001 were all significant differences.

To clarify the role of Bmp1 in the repair of damaged intestinal mucosa, intestinal epithelial cells were cultured to mimic the intestinal mucosal barrier. The expression of Bmp1 in IEC-6 or HIEC-6 was altered by the transfection of adenovirus. The effect of the transfection on Bmp1 expression was confirmed by qRT-PCR analysis (Fig. 2A). The CCK-8 assay was performed to monitor cell proliferation and the result showed that the proliferation of Bmp1-knockdown IEC-6 or HIEC-6 cells was significantly decreased, while overexpression of Bmp1 could significantly promote the proliferation of cells (Fig. 2B). Furthermore, the colonies remarkably increased in IEC-6 or HIEC-6 cells after Bmp1 transfection, while decreased significantly after si-Bmp1 transfection (Fig. 2C). Wound-healing assays verified the relationship between Bmp1 and cell migration in IEC-6 or HIEC-6 cells. The results showed that Bmp1 can promote cell migration, while knockdown of Bmp1 inhibits cell migration (Fig. 2D). The western blot results showed that the MMP9 content in Bmp1-knockdown cells decreased significantly, while the contents of E-cadherin and ZO-1 increased significantly. On the other hand, MMP9 expression increased and the expression of E-cadherin and ZO-1 decreased in the Bmp1 overexpression group compared with the control group (Fig. 2E). The results of the immunofluorescence assay showed that the E-cadherin content in Bmp1 knockdown cells increased significantly, while the content of E-cadherin decreased significantly in the Bmp1 group compared with the control group (Fig. 2F).

### Bmp1 has a protective effect on the intestinal tract of burned mice

To identify the underlying effects of Bmp1 on the burned mice, RNA samples were collected for detecting expression changes in Bmp1.

**Fig. 3:**
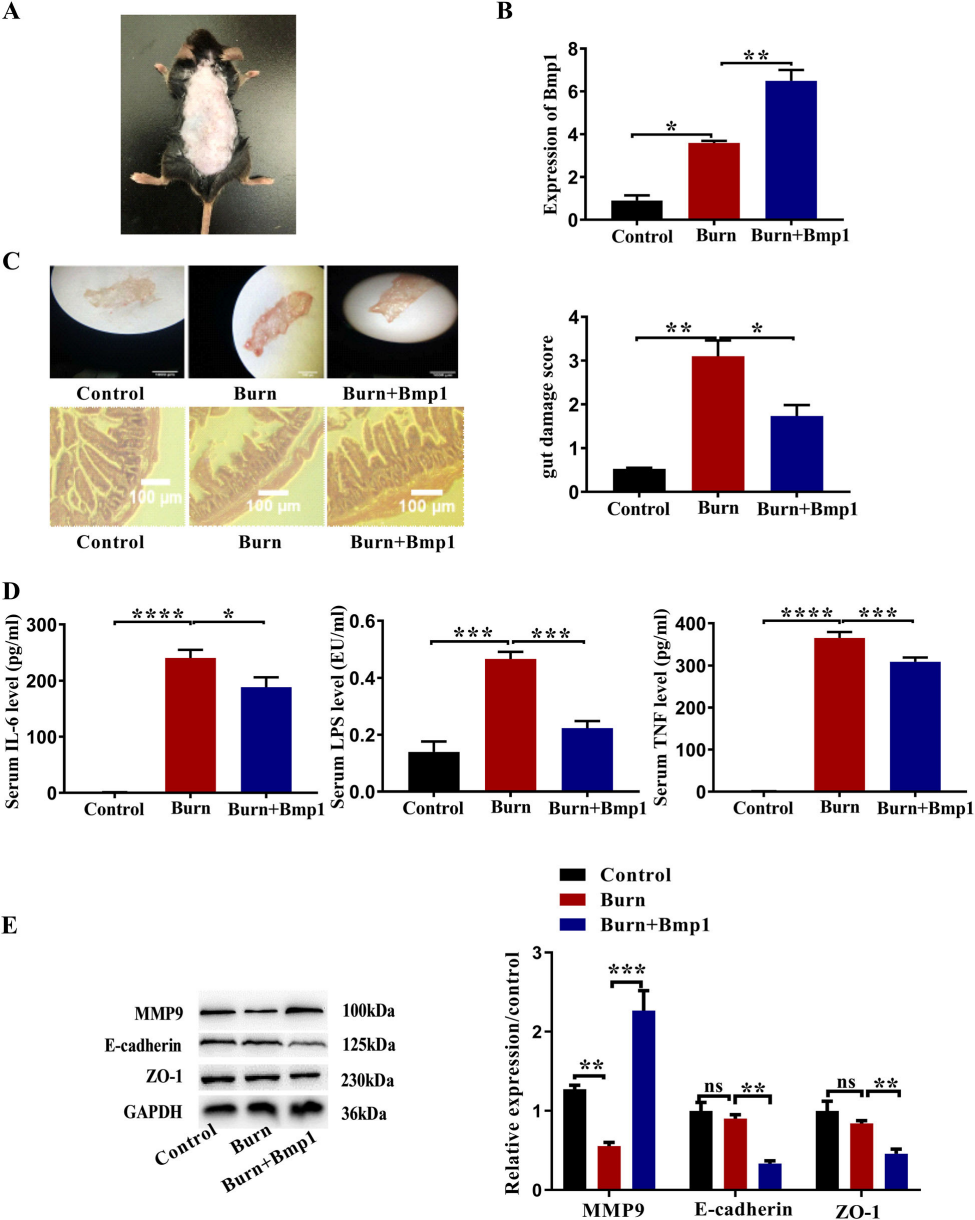
Overexpression of Bmp1 reduces intestinal mucosal injury in burned mice. **A** The third-degree mouse burn model was established. **B** Expression of Bmp1 was determined by qRT-PCR (*n* = 10). **C** Intestinal mucosa of burned mice were observed under a magnifying glass and intestinal mucosal injury scores were calculated by histological examination of H&E-stained sections under light microscopy (*n* = 10). **D** TNF-α, IL-6, and LPS (*n* = 10), **E** MMP9, E-cadherin, and ZO-1 were detected using ELISA and western blot, respectively (*n* = 10). All data were demonstrated as mean ± SD from three independent experiments performed in duplicate. *p < 0.05, **p < 0.01, ***p < 0.001 and ****p < 0.0001 were all significant differences.

Firstly, we established the third-degree mouse burn model (Fig. 3A) and verified the expression levels of Bmp1 in each group by qRT-PCR (Fig. 3B). Histological examination of H&E-stained sections under light microscopy showed that the intestinal mucosal injury in the Burn+Bmp1 group was slighter than that in the burn group (Fig. 3C, Table S1, and Table S2). The results of the ELISA showed that the levels of serum LPS, IL-6, and TNF-α in the burn group were significantly higher than those in the control group while the levels in the Burn+Bmp1 group were intermediate between the two groups (Fig. 3D). The western blot results showed that, compared with the control group, the expression of MMP9 in the Burn+Bmp1 group increased significantly, while the expression of E-cadherin and ZO-1 decreased significantly (Fig. 3E).

### Bmp1 serves as a miRNA sponge for miR-128-3p

**Fig. 4:**
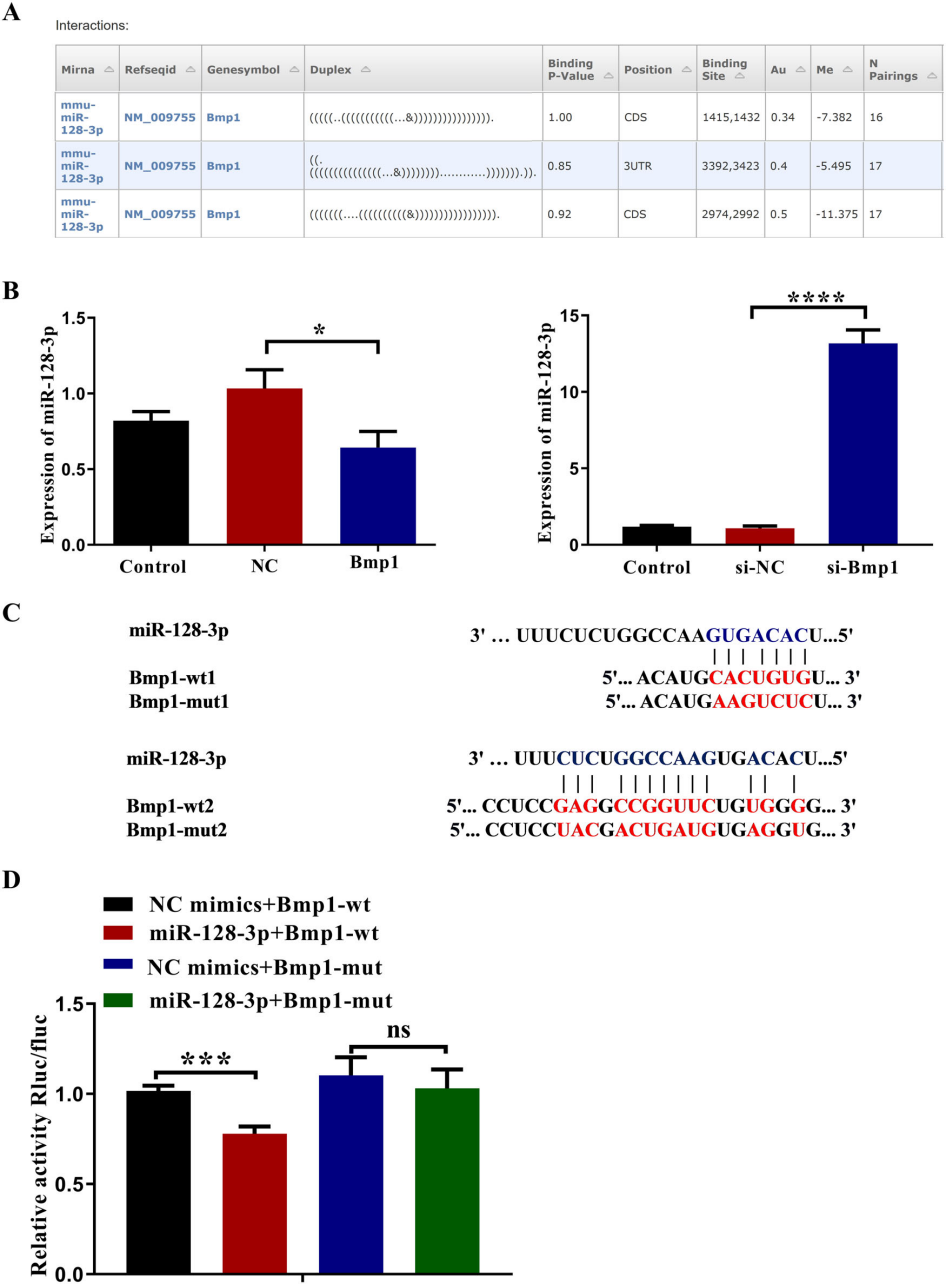
Bmp1 functions as an endogenous sponge of miR-128-3p. **A** The predicted sequence between Bmp1 and miR-128-3p using miRWalk. **B** Expression of miR-128-3p was determined using qRT-PCR (*n* = 3). **C** A schematic drawing indicated the mutant binding sites of miR-128-3p with respect to Bmp1. **D** Luciferase reporter assay was performed in 293T cells cotransfected with miR-128-3p mimic or mimic NC and luciferase reporters constructed with Bmp1-wt or Bmp1-mut. The luciferase activity was normalized to the ratio of firefly to the renilla activity (*n* = 3). All data were demonstrated as mean ± SD from three independent experiments performed in duplicate. *p < 0.05, **p < 0.01, ***p < 0.001 and, ****p < 0.0001 were all significant differences.

Studies have shown that lncRNAs can be used as ceRNAs to sponge miRNAs, leading to the de-expression of miRNA targets. To investigate the mechanism underlying Bmp1’s role in cell proliferation and migration, we used bioinformatic analysis to predict the potential miRNAs that may bind to Bmp1. Analysis with miRWalk software showed that miR-128-3p was predicted to bind to Bmp1 (Fig. 4A). qRT-PCR results proved that Bmp1 negatively regulated the expression of miR-128-3p (Fig. 4B). To further determine whether Bmp1 directly regulates miR-128-3p, we cloned the hypothetical miR-128-3p target-binding sequence into a luciferase construct. The miR-128-3p mimic and Bmp1 wild-type or mutant reporter genes were transfected into 293T cells (Fig. 4C). It was found that the miR-128-3p mimic significantly reduced luciferase activity of the Bmp1 wild-type reporter gene, and the mutation of the Bmp1 target successfully reversed the previous inhibition (Fig. 4D). These findings indicate the binding conditions of miR-128-3p and Bmp1.

### Upregulation of miR-128-3p alters the impact of Bmp1 overexpression on IEC-6 cells

**Fig. 5:**
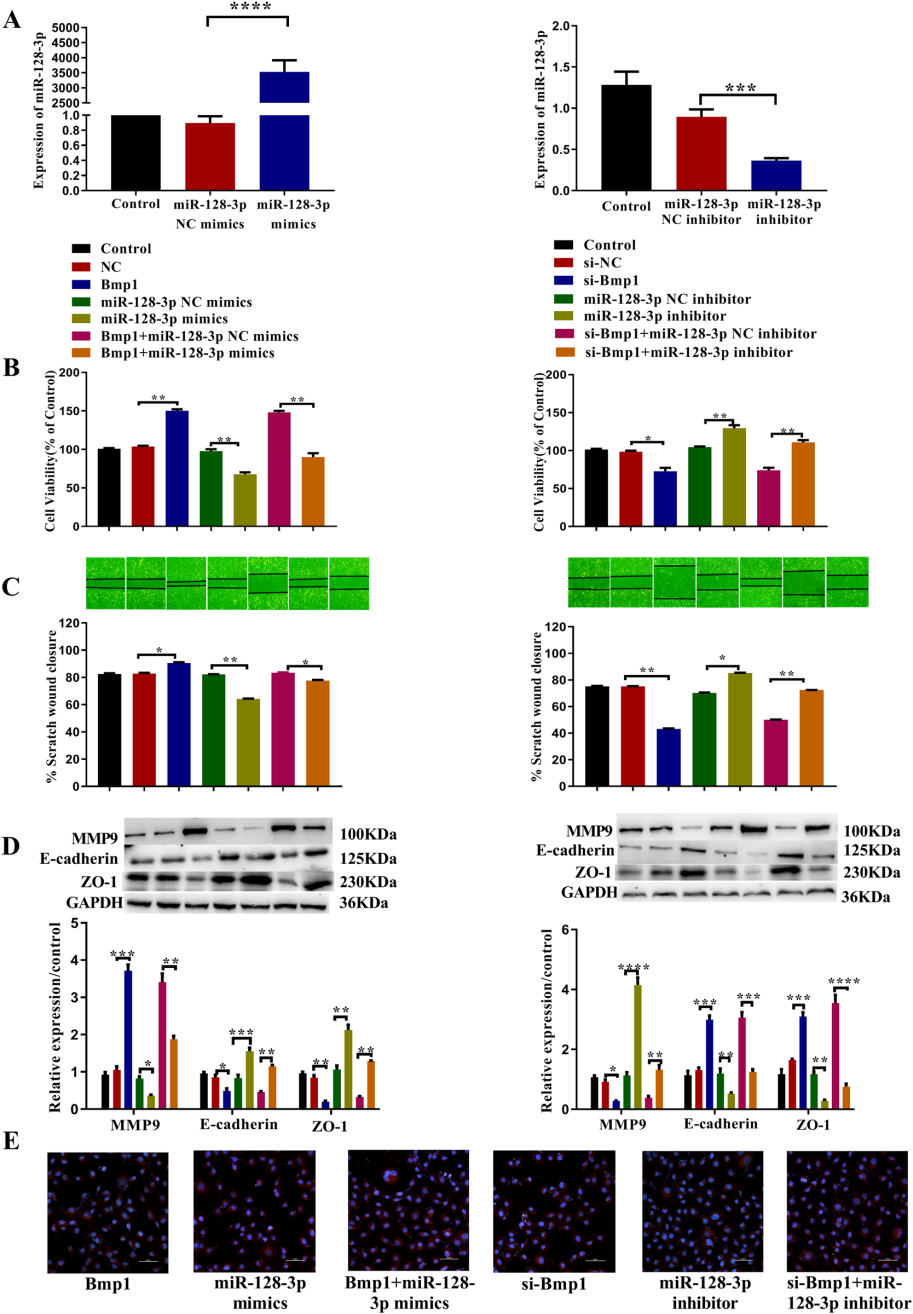
MiR-128-3p mimic can rescue the impacts of overexpression of bmp1 on IEC-6 cells. **A** The expression of miR-128-3p in IEC-6 cells transfected with miR-128-3p mimic or miR-128-3p inhibitor were determined using qRT-PCR (*n* = 3). **B** Cell viability (*n* = 3), **C** cell migration ability (*n* = 3), **D** E-cadherin and ZO-1 were analyzed using cell counting kit-8 assay, wound healing assay and western blot, respectively (*n* = 3). **E** Representative immunofluorescence analysis showed the content of E-cadherin in treated IEC-6 cells (*n* = 3). All data were demonstrated as mean ± SD from three independent experiments performed in duplicate. *p < .05, **p < .01, ***p < .001 and ****p < .0001 were all significant differences.

To further investigate how miR-128-3p was involved in Bmp1-mediated cell proliferation and migration, rescue assays were performed. As shown in Fig. 5A, the miR-128-3p expression was upregulated or suppressed after transfection with miR-128-3p mimic or miR-128-3p inhibitor, respectively. The CCK-8 results showed that overexpression of Bmp1 and knockdown of miR-128-3p could significantly promote cell proliferation, while knockdown of Bmp1 and overexpression of miR-128-3p could significantly inhibit cell proliferation. When Bmp1 and miR-128-3p were overexpressed or knocked down at the same time, miR-128-3p could reverse the effects of Bmp1 on cell proliferation (Fig. 5B). The wound-healing assay and western blot results showed that overexpression of Bmp1 and knockdown of miR-128-3p promoted both cell migration and the expression of MMP9, and reduced the expression of E-cadherin and ZO-1. After knocking down Bmp1 and over-expressing miR-128-3p, IEC-6 cell migration was inhibited, MMP9 concentrations were significantly reduced and the expression of E-cadherin and ZO-1 were significantly increased. When Bmp1 and miR-128-3p were overexpressed or knocked down at the same time, miR-128-3p reduced the effect of Bmp1 on cell migration and migration-related proteins MMP9, E-cadherin, and ZO-1 (Fig. 5C, D). Immunofluorescence assay results showed that overexpression of Bmp1 and knockdown of miR-128-3p significantly reduced the expression of E-cadherin, while E-cadherin levels increased on Bmp1 knockdown and miR-128-3p overexpression. When Bmp1 and miR-128-3p were overexpressed or knocked down at the same time, miR-128-3p reduced the effect of Bmp1 on E-cadherin (Fig. 5E).

### *Phf6* is targeted by miR-128-3p

**Fig. 6:**
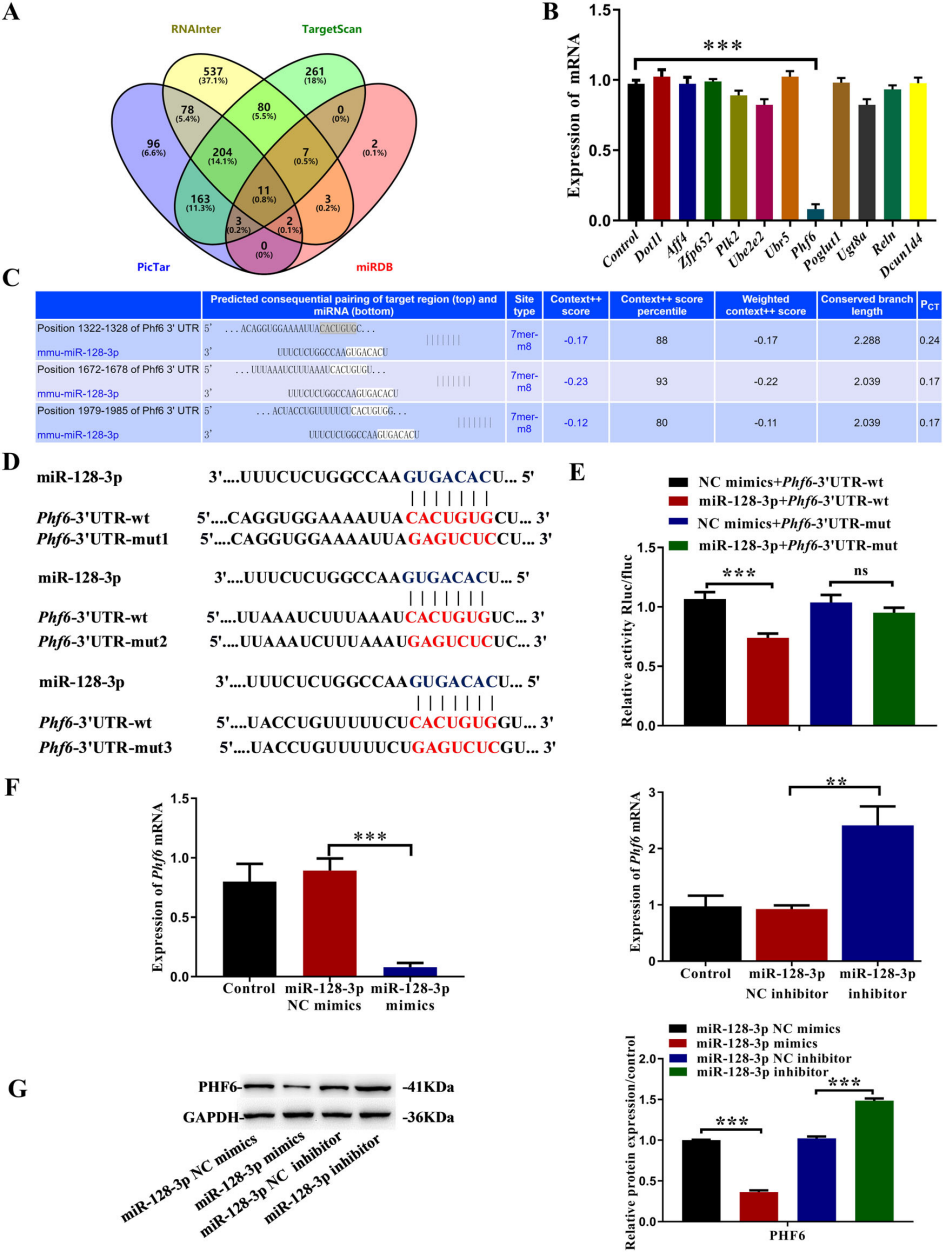
*Phf6* is the target gene of miR-128-3p. **A** A Venn diagram indicated that 30 genes may be functional targets of miR-128-3p. **B** qRT-PCR showed that miR-128-3p dramatically reduced the mRNA expression level of *Phf6*. **C** The predicted sequence between miR-128-3p and *Phf6* using TargetScanMouse. **D** The mutant binding sites of miR-128-3p at the 3′ UTR of *Phf6* mRNA. **E** Relative luciferase activities of *Phf6*−3′ UTR-wt or *Phf6*−3′ UTR-mut were analyzed in HEK293T cells after co-transfection with miR-128-3p mimic or mimic NC. The luciferase activity was normalized to the ratio of firefly to the renilla activity (*n* = 3). **F**, **G** qRT-PCR and western blot analysis were used to detect the PHF6 expression in IEC-6 cells transfected with miR-128-3p mimic and miR-128-3p inhibitor (*n* = 3). All data were demonstrated as mean ± SD from three independent experiments performed in duplicate. *p < 0.05, **p < 0.01, ***p < 0.001, and ****p < 0.0001 were all significant differences.

Because lncRNA, as competitive ceRNAs, compete with mRNA for miRNA binding^20^, we searched four bioinformatics tools (miRDB, PicTar, TargetScan, and RNAInter) and jointly predicted that 11 genes may be biological targets of miR-128-3p (Fig. 6A). Through qRT-PCR, we identified *Phf6* as our research object (Fig. 6B). We performed a bioinformatic analysis using TargetScan and identified the potential binding sites between miR-128-3p and *Phf6* (Fig. 6C). To verify whether *Phf6* is indeed targeted by miR-128-3p, we further constructed the reporter gene vectors *Phf6*-3′ UTR-wild and *Phf6*-3′ UTR-mut (Fig. 6D). After briefly co-transfecting 293T cells with miR-128-3p mimics/NC mimic and the reporter gene vector *Phf6*−3′UTR-wild/mut, the cells were assayed for dual-luciferase reporter genes. In the *Phf6*-3′ UTR-wild transfection group, treatment with the miR-128-3p mimic reduced the relative activity of luciferase while this did not occur in the group transfected with *Phf6*-3′UTR-mut (Fig. 6E). This indicates that miR-128-3p reduces *Phf6* expression by binding to *Phf6* 3’UTR. qRT-PCR showed that the miR-128-3p mimic decreased *Phf6* levels significantly while miR-128-3p inhibitors significantly increased *Phf6* levels (Fig. 6F). Western blotting also confirmed that miR-128-3p inhibited the expression of PHF6 protein in IEC-6 cells (Fig. 6G). These results confirm that *Phf6* is a direct target of miR-128-3p.

### PHF6 rescues the effect of miR-128-3p

**Fig. 7:**
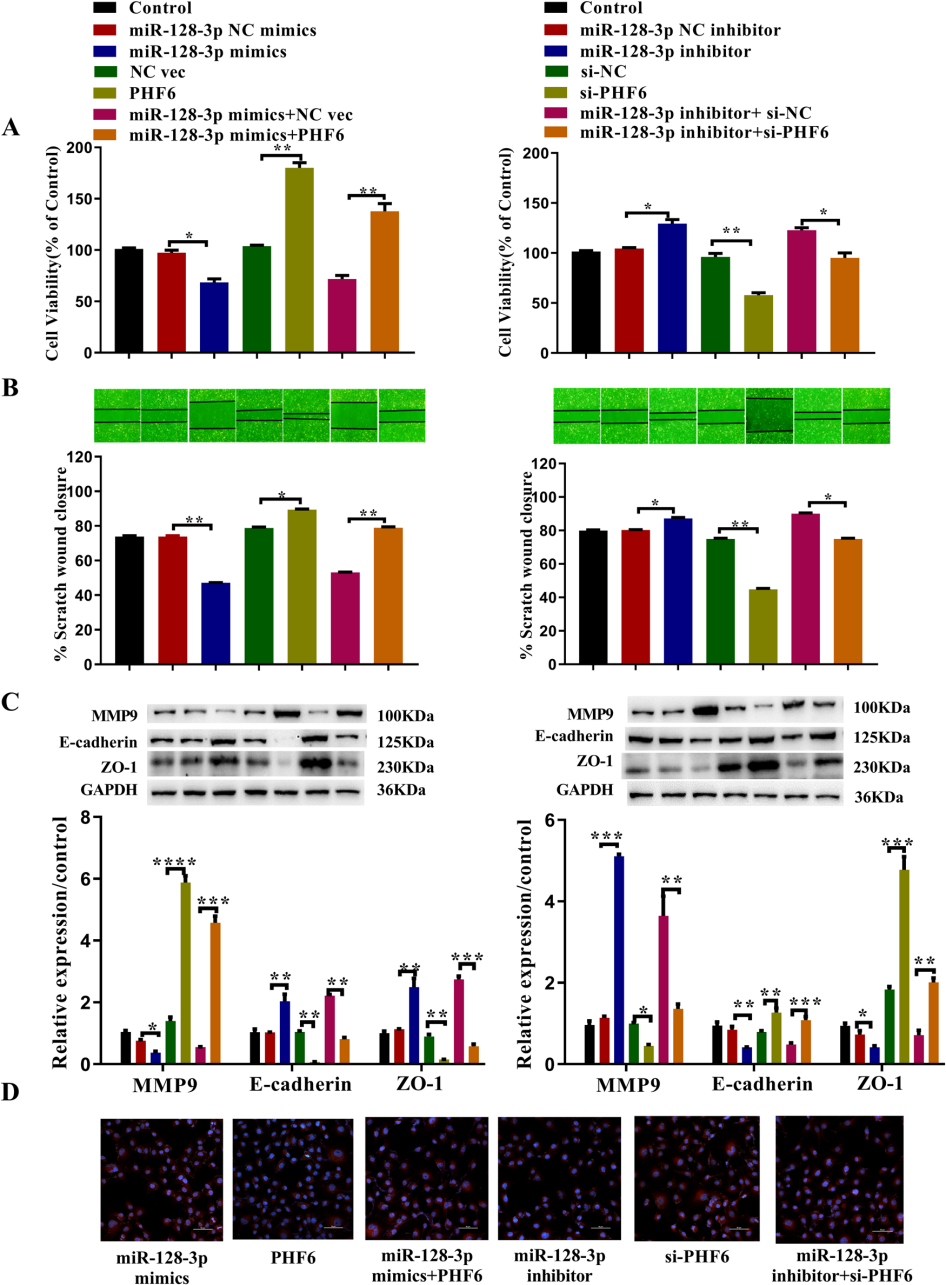
Overexpression of PHF6 can rescue the effect of miR-128-3p mimic on IEC-6 cells. **A** Cell viability (*n* = 3), **B** cell migration ability (*n* = 3), **C** MMP9, E-cadherin and ZO-1 were analyzed using cell counting kit-8 assay, wound healing assay, and western blot, respectively (*n* = 3). **D** Representative immunofluorescence analysis showed the content of E-cadherin in treated IEC-6 cells (*n* = 3). All data were demonstrated as mean ± SD from three independent experiments performed in duplicate. *p < 0.05, **p < 0.01, ***p < 0.001, and ****p < 0.0001 were all significant differences.

To further investigate the involvement of PHF6 in miR-128-3p-mediated cell proliferation and migration, we performed rescue assays. The CCK-8 results showed that overexpression of PHF6 and knockdown of miR-128-3p significantly promoted cell proliferation while knockdown of PHF6 and overexpression of miR-128-3p inhibited proliferation. When miR-128-3p and PHF6 were overexpressed or knocked down at the same time, PHF6 reduced the effect of miR-128-3p on cell proliferation (Fig. 7A). Wound-healing assay and western blot results showed that overexpression of PHF6 and knockdown of miR-128-3p could significantly promote cell migration, promote the expression of MMP9, and significantly reduce the expression of E-cadherin and ZO-1. Knockdown of PHF6 and overexpression of miR-128-3p inhibited both cell migration and MMP9 expression, while increasing the expression of E-cadherin and ZO-1. When miR-128-3p and PHF6 were overexpressed or knocked down at the same time, PHF6 weakened the effect of miR-128-3p on cell migration and the migration-related proteins MMP9, E-cadherin, and ZO-1(Fig. 7B, C). Immunofluorescence assay results showed that overexpression of PHF6 and knockdown of miR-128-3p could significantly reduce the expression of E-cadherin. Knockdown of PHF6 and overexpression of miR-128-3p significantly increased the expression of E-cadherin. When miR-128-3p and PHF6 were overexpressed or knocked down at the same time, PHF6 reduced the effect of miR-128-3p on E-cadherin (Fig. 7D).

### PHF6 plays a physiological role in IEC-6 cells possibly by activating PI3K/AKT signaling

**Fig. 8:**
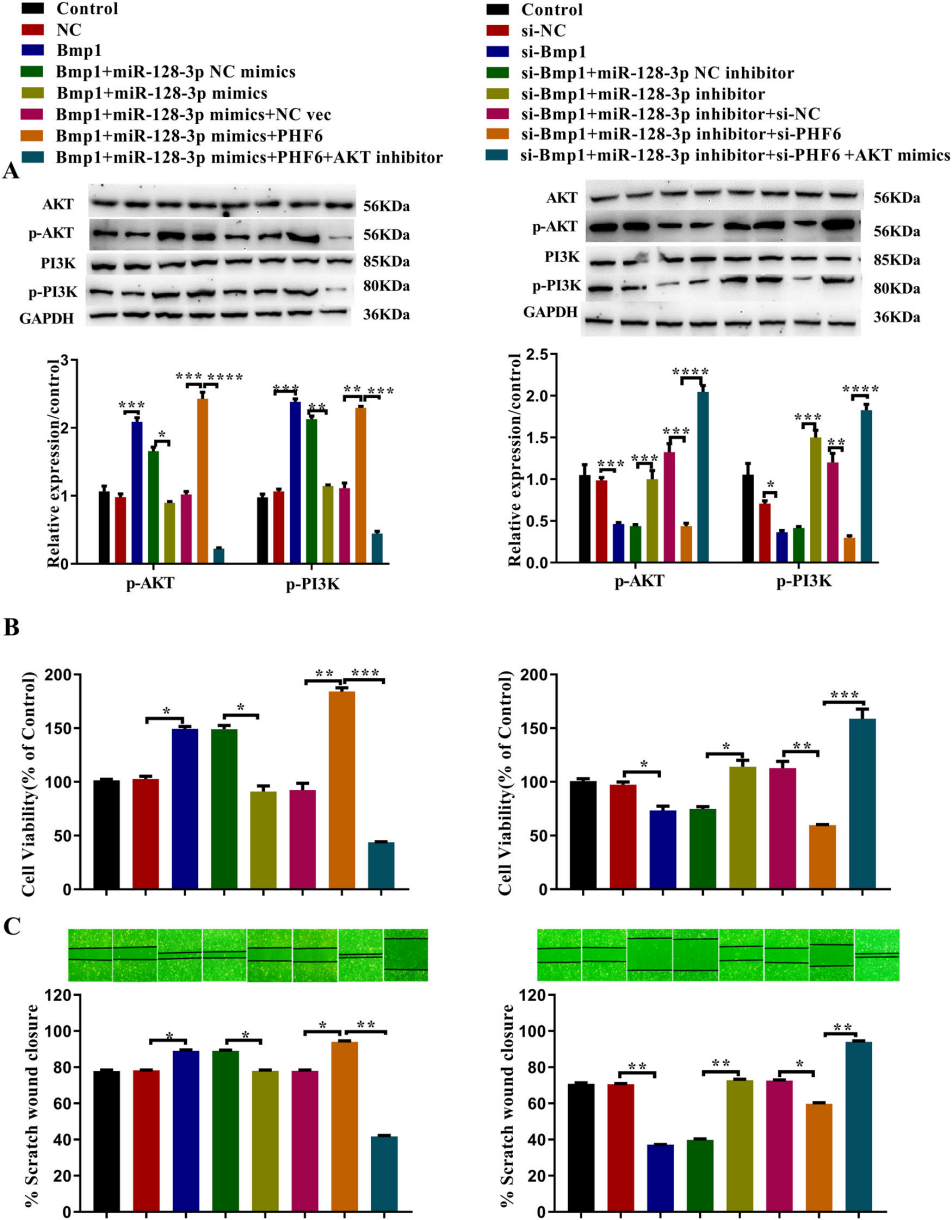
PHF6 promotes the proliferation and migration of IEC-6 cells by activating the PI3K/AKT signaling pathway. **A** The alter expression of PI3K, p-PI3K,AKT, p-AKT in treated IEC-6 cells (*n* = 3), **B** cell viability (*n* = 3), **C** cell migration ability were analyzed using western blot, cell counting kit-8 assay, and wound healing assay, respectively (*n* = 3). All data were demonstrated as mean ± SD from three independent experiments performed in duplicate. *p < 0.05, **p < 0.01, ***p < 0.001 and ****p < 0.0001 were all significant differences.

Western blot results showed that overexpression of Bmp1 and PHF6 could activate the PI3K/AKT signaling pathway, while knockdown of Bmp1 and PHF6 inhibited the pathway. When Bmp1 and miR-128-3p were overexpressed or knocked down at the same time, miR-128-3p reduced the effect of Bmp1 on PI3K/AKT activation. When Bmp1, miR-128-3p, and PHF6 were overexpressed or knocked down at the same time, PHF6 reduced the effect of miR-128-3p and Bmp1 on the pathway. Interference with AKT expression altered the effects of PHF6 (Fig. 8A). The CCK-8 results showed that overexpression of Bmp1 and PHF6 promoted cell proliferation, while knockdown of both inhibited proliferation. At the same time, when Bmp1 and miR-128-3p were overexpressed or knocked down, miR-128-3p reduced Bmp1’s promotion of cell proliferation. When Bmp1, miR-128-3p, and PHF6 were overexpressed or knocked down at the same time, PHF6 reduced the effects of both miR-128-3p and Bmp1 on cell proliferation. Interference with AKT expression altered PHF6’s effects on proliferation (Fig. 8B). The wound-healing assay showed that overexpression of Bmp1 and PHF6 could significantly promote cell migration, while knockdown of Bmp1 and PHF6 reversed this effect. However, when Bmp1 and miR-128-3p were overexpressed or knocked down, miR-128-3p could reverse the effects of Bmp1 on cell migration. When Bmp1, miR-128-3p, and PHF6 were overexpressed or knocked down at the same time, PHF6 could weaken the effects of miR-128-3p and Bmp1 on cell migration. Interference with the AKT expression level altered the effects of PHF6 on cell migration (Fig. 8C).

## Discussion

Burns can change the structure and function of the intestinal tract and damage the intestinal mucosal barrier, resulting in intestinal bacterial translocation and severe burn infections^21,22^. Therefore, protecting the integrity of the intestinal structure after burns and promoting the recovery of the intestinal mucosa have important clinical significance. This study shows that the content of Bmp1 is upregulated after intestinal mucosal injury. The overexpression of Bmp1 has a protective effect on the intestine of burned mice and can significantly improve the proliferation and migration ability of IEC-6 or HIEC-6 cells through the lncRNA Bmp1/miR-128-3p/PHF6/PI3K/AKT pathway.

LncRNAs perform important functions in the regulation of many biological processes, including gene transcription and translation. Abnormal expression of lncRNAs occurs in many diseases. In this study, we found that the expression level of Bmp1 in the mouse intestine increased significantly at 24 h after the burn, while the intestinal mucosal injury was severe, and the expression level of the migration-related protein MMP9 was significantly decreased. To verify the physiological role of Bmp1, we used IEC-6 or HIEC-6 cells to build an intestinal mucosal injury model in vitro. The results showed that Bmp1 could significantly promote the proliferation and migration of the cells, as well as the expression level of migration-related proteins such as MMP9. In addition, we also verified the role of Bmp1 in the burn mouse model. The intestinal mucosal injury of burned mice was significantly alleviated after intragastric injection of Bmp1, and the injury index was significantly lower than that of the burn group.

Although Bmp1 has been shown to be involved in the repair of damaged intestinal mucosa, the exact molecular mechanism is unclear. Recently, the “ceRNA” hypothesis has been proposed: that lncRNA functions by acting as a sponge for miRNA. Binding between lncRNAs and miRNAs blocks the inhibitory effects of miRNAs on mRNA^23^. For example, the long-chain non-coding RNA LNC-SEMT regulates IGF2 expression through the sponge miR-125b, which promotes the development and growth of sheep muscle^24^. LncRNA lnc-HC regulates PPARγ- mediated lipid metabolism through miR-130b-3p^25^. To explore the mechanism of intestinal mucosal repair mediated by Bmp1, bioinformatics analysis was performed and the results showed that miR-128-3p can bind to Bmp1. Then, we further explored the effect of miR-128-3p on the proliferation and migration of IEC-6 cells and the relationship between miR-128-3p and Bmp1. First, our results verified that miR-128-3p negatively regulates the proliferation and migration of IEC-6 cells: miR-128-3p overexpression downregulates the expression of MMP9 and inhibits cell migration, whereas the miR-128-3p inhibitor upregulates MMP9 and promotes cell migration. Second, Bmp1 negatively regulates the expression of miR-128-3p: overexpression of Bmp1 reduces the expression of miR-128-3p and inhibits Bmp1 upregulation of miR-128-3p expression. To determine whether there is direct binding between Bmp1 and miR-128-3p, we performed a luciferase reporter gene assay which showed that miR-128-3p inhibits the luciferase activity of the vector containing the Bmp1 sequence. Mutation of the hypothetical miR-128-3p target site reversed this inhibition, indicating that Bmp1 binds directly to miR-128-3p via the hypothetical MRE in the “ceRNA” regulatory network.

We used bioinformatics analysis to identify a potential *Phf6* binding site on miR-128-3p, suggesting that *Phf6* is targeted by miR-128-3p. To verify our bioinformatics analysis, qRT-PCR and western blot were used, showing that IEC-6 cells transfected with miR-128-3p mimic downregulated PHF6 expression, while transfection with the miR-128-3p inhibitor increased PHF6 expression. To determine whether miR-128-3p binds directly to *Phf6*, luciferase reporter gene analysis was carried out. MiR-128-3p inhibited luciferase activity of the vector containing the *Phf6* sequence. The putative mutation of the target site of miR-128-3p reversed this inhibitory effect, indicating that, in this “ceRNA” regulatory network, miR-128-3p binds directly to *Phf6* through the putative MRE. All these data indicate that miR-128-3p targets the *Phf6* gene. To further verify whether Bmp1 regulates the process of proliferation and migration of IEC-6 cells by regulating the expression of PHF6 through the sponge miR-128-3p, we carried out remedial experiments. The results showed that the increased expression of miR-128-3p reverses Bmp1’s promotion of cell proliferation and migration, and upregulation of PHF6 reversed the inhibitory effect of miR-128-3p on the function of Bmp1. The PI3K/AKT signaling pathway plays an important role in cell regulation such as cell proliferation and migration^26^. Therefore, the interaction between PI3K/AKT pathway and Bmp1 was evaluated. We found that overexpression of Bmp1 could activate thePI3K/AKT signaling pathway, upregulation of miR-128-3p reversed the effect of Bmp1 on the pathway, and upregulation of PHF6 reversed the inhibitory effect of miR-128-3p on the pathway. To sum up, these results show that Bmp1 promotes the epigenetic transcription of PHF6 by interacting with miR-128-3p, thus promoting the activation of the PI3K/AKT signaling pathway.

In conclusion, our data revealed that high expression of Bmp1 may promote the repair of damaged intestinal mucosa through ceRNA mediation and regulation of PHF6 expression by sponging miR-128-3p. The PI3K/AKT signaling may be a downstream factor in mediating the impact of Bmp1 on injured intestinal mucosa(Fig S1). The Bmp1-miR-128-3p-PHF6-PI3K/AKT axis may prove a promising approach for the diagnosis and treatment of damaged intestinal mucosa.

## Materials and methods

### Experimental animals, burn model, tissue, and blood processing

Healthy, clean, adult, C57BL/6J mice (20–30 g; *n* = 50) were used in this study. This work was approved by the Animal Ethics Committee of Xuzhou Medical University, China on September 15, 2016. Mice were raised adaptively for one week under conditions of constant temperature and ventilation and were fasted with free water availability for 12 h before the experiment. The mice were anesthetized by intraperitoneal injection of 1 g/L pentobarbital sodium (40 mg/kg). The fur on the back was removed with scissors and hair removal cream, exposing 30% of the body. After the bare skin was soaked in water at 100 °C for 8 s, 50 mL/kg of lactic acid was injected intraperitoneally, and the mice were given anti-shock treatment. This treatment had previously been shown to cause full-thickness burns. Mice were randomly divided into three groups: control group (C, *n* = 10), burn group (B, *n* = 10), and burn + Bmp1 group (*n* = 10). Bmp1 adenovirus (Shanghai Hanheng Bio-Technology, China) was administered to mice in the burn + Bmp1 group (10 µl/g), 1 h before severe burns, by intragastric injection. Blood and intestinal samples were collected at designated time points after burns. Blood samples were taken, centrifuged at 4 °C (12,000 × *g*, 20 min) to prepare serum, and enzyme-linked immunosorbent assay (ELISA) was used to detect lipopolysaccharide (LPS), interleukin-6 (IL-6), and tumor necrosis factor-α (TNF-α). A portion of each intestinal tissue specimen was stored at −80 °C for western blotting and real-time PCR quantification, and the remaining specimens were fixed in 10% formalin solution for hematoxylin-eosin (H&E) staining.

### ELISA

The ELISA kit for detecting LPS, IL-6, and TNF-α in mouse serum was purchased from Sigma Biotechnology Company (St Louis, MI, USA) and used according to the manufacturer’s instructions.

### Western blot analysis

The cells were lysed with RIPA lysis buffer and the total protein concentration was measured with a Protein Assay Kit (Beyotime, China). Separate equal amounts of protein were electrophoresed on SDS-PAGE and transferred to PVDF membranes (Amersham, USA). The membrane was blocked with 5% BSA at room temperature for 2 h and incubated with the primary antibody overnight. The primary antibodies for PHF6, PI3K, p-PI3K, p-AKT, and AKT were obtained from Abcam (Cambridge, MAUSA) and used at 1:800 dilution. MMP9, E-cadherin, and ZO-1 antibodies were purchased from Proteintech (Rosemont, IL, USA) and used at 1:10,000 dilution. Anti-GAPDH (BioWorld, Dublin, OH, USA) at 1:10,000 dilution was used as the loading control. The membrane was washed three times with Tris-buffered saline containing 0.05% Tween 20 (TBST) for 5 min each time. Membranes were then incubated with secondary antibody (1:10000, Proteintech, Rosemont, IL, USA) at room temperature for 1 h and washed with TBST for 30 min. Finally, Odyssey infrared imaging software Image Studio Version 5.2 (Li-Cor Biotechnology, Bad Homburg, Germany) was used to record the images. The western blot analysis used Image J software for quantitative analysis.

### Intestinal mucosa morphology

The mice were killed, the intestines were removed, cut along the longitudinal axis, rinsed with isotonic saline, and flattened. We observed the mucosal damage under a magnifying glass. The view of each sample was divided into five sections and each section was scored separately. These five scores were then added to provide a final damage score for the sample. The scoring method followed a previous study^27^ and the scoring system is presented in Table S1.

### H&E staining

The intestinal tissue was preserved with 10% formalin solution for pathological sectioning (thickness 3 µm). We used the method described by Perez-Chanona et al. to assess the state of intestinal injury^28^. The scoring system is presented in Table S2.

### Cell culture

The rat intestinal epithelial cell (IEC-6) line was obtained from the Shanghai Institute of Biological Sciences (Shanghai, China) and was cultured in RPMI-1640 with 10% fetal bovine serum (FBS) (Zhejiang Tianhang Biotechnology Co., Ltd., Zhejiang, China) at 37 °C and 5% carbon dioxide. The media were supplemented with 100 U penicillin and 100 µg streptomycin per ml. The human intestinal epithelial cells (HIEC-6) used in this study were purchased from ATCC (Manassas, VA, USA). HIEC-6 cells were cultured at 37 °C and 5% CO_2_ in high-glucose DMEM containing 5% FBS, 10 mM HEPES, 4 mM glutamine, 50 U/ml penicillin, 50 mg/ml streptomycin, 5 mg/ml epidermal growth factor, and 100 ml/ml insulin, transferrin, and selenium.

### Adenovirus infection

The recombinant adenovirus and scrambled control (NC) were obtained from the Shanghai Hanheng Company. The GeneBank accession number of Bmp1 is NR_033241.1. Cells were transfected by adenovirus (MOI = 200) exposure in 8 mL RPMI-1640 supplemented with 10% FBS for 24 h. The infected cells were cultured in a conventional medium, and the expression level of Bmp1 was detected.

### Transfection of miRNA mimic/ inhibitor

MiRNA mimic/inhibitor were obtained from Shenggong Company (Shanghai, China). After culturing to 90% confluence, 6-well plates were inoculated with 2 × 10^5^ cells per well. The miRNA mimic/ miRNA NC mimic (50 nmol /L), or miRNA inhibitor/miRNA NC inhibitor (20 nmol/L) were transfected into the cells using X-tremeGENE siRNA Transfection Reagent (Roche, Switzerland). After incubation for 48 h, the cell proteins and the total RNA were extracted from the cells, and expression of the endogenous miR-128-3p was detected using qRT-PCR.

### siRNA transfection

Cells were transfected with siRNA reagents using X-tremeGENE siRNA Transfection Reagent (Roche, Switzerland) according to the manufacturer’s protocol. The pooled siRNAs for knocking down the expression of Bmp1(20nmol/L), PHF6 (20 nmol/L), and the negative control (NC) were purchased from Hanheng Company (Shanghai, China). After transfection for 72 h, the expression of Bmp1 and PHF6 mRNA was assessed by qRT-PCR.

### Transfection of PHF6 overexpression plasmid

The PHF6 overexpression plasmid was obtained from Hanheng Company (Shanghai, China). The IEC-6 cells were seeded in 6-well plates (2 × 10^5^ cells per well). The PHF6 plasmid (50 nmol/L) was transfected into the cells using LipoFiter 3 (Hanheng, Shanghai, China). After incubation for 72 h, the expression of PHF6 was measured using qRT-PCR and Western blot.

### AKT mimic (SC79) and inhibitor (ly294002) transfection

#### AKT mimic transfection

Five milligrams of AKT mimic (Beyotime company, Nanjing, China) were added to 1.37 ml DMSO, to prepare 10 mM solution. One day before transfection, 2 × 10^5^ cells were inoculated into each well of a 6-well plate and the culture medium replaced with fresh cell culture medium without phenol red. After 24 h of culture, the previously configured solution (8 µg/ml) was added and cultured overnight. The follow-up experiment was carried out 20 h later.

#### AKT inhibitor transfection

The day before transfection, 2 × 10^5^ cells per well were inoculated in 6-well plates and grown until 50%–70% confluent. After changing to fresh cell culture medium, the final concentration of the AKT inhibitor (APE×BIO, USA), was 10 µM. After gently mixing, the follow-up experiment was carried out after incubation for 24 h.

### Quantitative real-time PCR analysis of lncRNA, miRNA, and mRNA levels

Real-time PCR was used to quantify Bmp1, miR-128-3p, and PHF6. Total RNA of tissues and cells was extracted by Trizol (Invitrogen, CA, USA). Reverse transcription was performed using miRcute miRNA first-strand cDNA synthesis kit (Tiangen Biotechnology, Beijing, China) according to the manufacturer’s instructions. The internal references were GAPDH or U6. Real-time fluorescent quantitative PCR was performed using the LightCycler 480 system (Roche, Basel, Switzerland) and Fast Universal SYBR Green Master Mix (Roche). In the melting curve analysis, there is only one peak per PCR product. The abundance of mRNA was calculated by the 2^−∆∆Ct^ method, considering the gene-specific efficiency, and normalized to the above average expression index.

The primers used in this experiment are listed in Table S3.

### Cell counting kit-8 (CCK-8) assay

After transfection for 24 h, the cells were digested, collected, and seeded into 96-well plates at 2 × 10^3^ per well. Each group was set up with six replicates. The CCK-8 method (Dojindo, Kumamoto, Japan) was used to measure the cell survival rate at different time points. Ten microliters of CCK-8 solution was added to each well 2 h before the assay and incubated at 37 °C for 1 h. The absorbance of each well at 450 nm was measured with a microplate reader (Thermo Fisher Scientific, Waltham, MA, USA).

### Colony formation assay

After 24 h transfection, cells were plated onto a 6-well plate at a density of 500 cells per well and cultured for consecutive 10 days until colony formation. Then the cells were washed with PBS, fixed in methanol for 30 min and stained with 0.1% crystal violet dye. The colonies (>50 cells per colony) were observed and counted under a light microscope. All assays were performed in triplicate.

### Wound-healing assay

The cells were seeded in 6-well plates at a density of 2 × 10^5^ cells/ml and incubated until they reached 90–100% confluence. Subsequently, an artificial wound was carefully made using a sterile P-10 pipette tip. The cells were thereafter treated differently for an additional 24, 48, or 72 h in serum-free medium. The rate of cell migration into the wound area was examined and photographed. The degree of wound contraction in a single group was quantified by the percentage of the area of cells that had migrated into the wound relative to the total area of the wound. Experiments were repeated three times.

### Immunofluorescence assay

Immunofluorescence assay was used to detect E-cadherin expression in cells. After different treatments, the cells were cultured on glass coverslips in 6-well plates and fixed with 4% paraformaldehyde for 10 min at room temperature. The cells were then permeabilized with 0.1% Triton X-100 for 10 min on ice. The cells were blocked with goat serum (DCS/BioGenex, Hamburg, Germany) for 60 min at room temperature and then incubated with the primary antibody (1:50, Proteintech) overnight at 4 °C. The cells were then incubated with FITC-tagged secondary antibodies (1:100, Huaan Company, China) for 60 min at room temperature. Subsequently, cells were washed and nuclei were counterstained with DAPI (Beyotime Company, China). Images were captured using a laser scanning confocal microscope (Olympus, Japan).

### Dual-luciferase reporter assay

The bioinformatic websites miRWalk and TargetScan were used to predict the binding sites between Bmp1 and miR-128-3p and between miR-128-3p and PHF6. The dual-luciferase reporter gene experiment was performed to verify whether miR-128-3p was the target gene of Bmp1 and *Phf6* was the target gene of miR-128-3p. Bmp1 transcripts (NR_033241.1) and *Phf6* 3′-UTR with or without mutations within miR-128-3p binding sites were cloned into the psiCHECK-2 luciferase reporter vector (Promega, Madison, WI, USA). The miR-128-3p mimic or negative control (NC) were cotransfected with the reporter plasmid for 48 h. The determination of the relative luciferase activity was done by the dual luciferase reporter gene detection system (Promega, Madison, WI, USA).

### Statistical analysis

Statistical analysis was performed using SPSS 19.0 software (IBM Corp., Armonk, NY, USA). Data are expressed as mean ± standard deviation (SD). Student’s *t*-test or one-way analysis of variance (ANOVA) was used to evaluate the differences between groups. *P* < 0.05 was considered statistically significant.

## Supplementary information

Figure S1

Table S1

Table S2

Table S3

## Data Availability

The data sets used and/or analyzed during the current study are available from the corresponding author on reasonable request.
