# Supplementary figures and images for: LncRNA Bmp1 promotes the healing of intestinal mucosal lesions via the miR-128-3p/PHF6/PI3K/AKT pathway
Source: Cell Death Dis. 2021 Jun 9;12(6):595. doi: 10.1038/s41419-021-03879-2 (PMC8190101; doi:10.1038/s41419-021-03879-2)

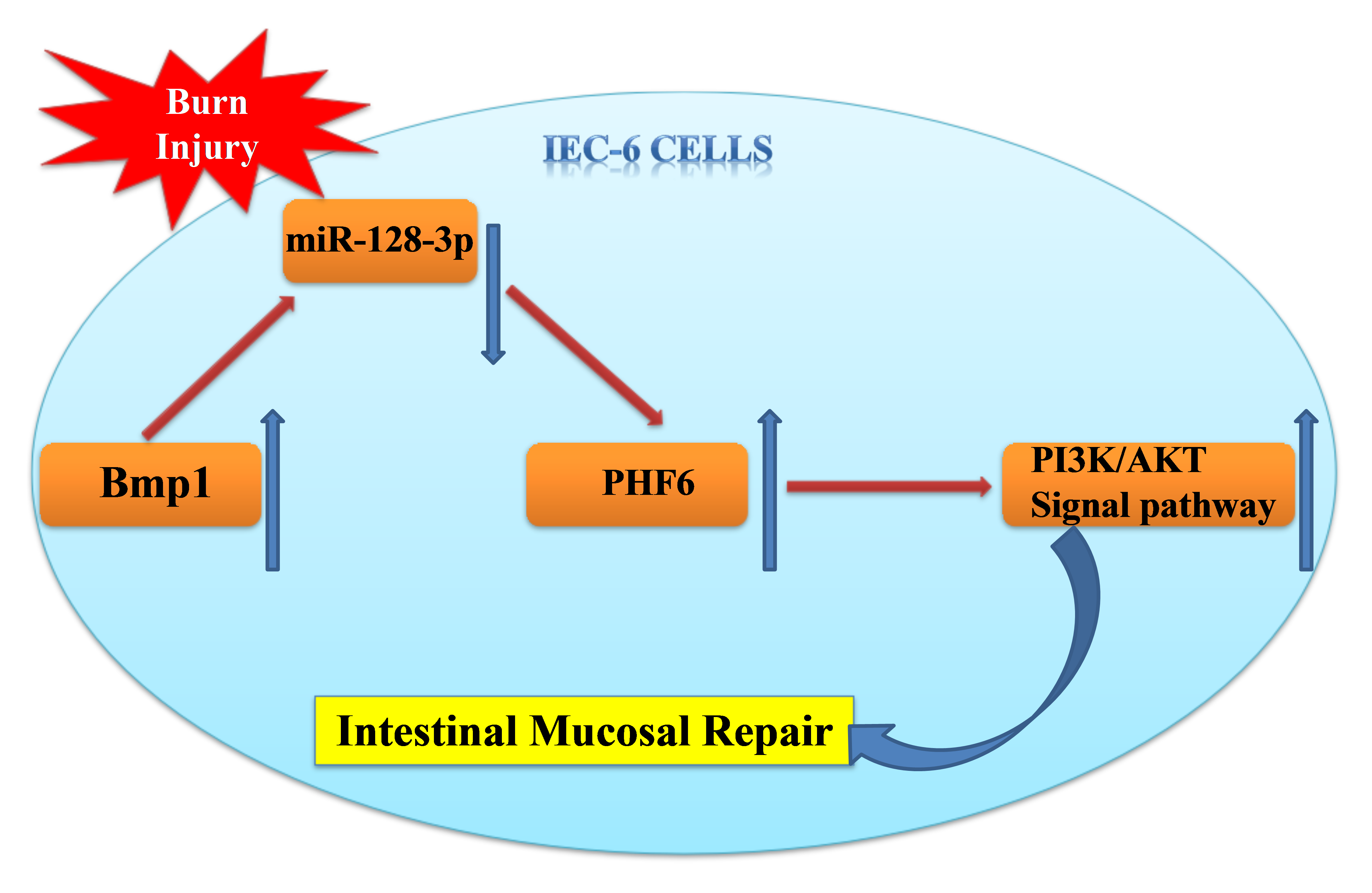

Supplement: Supplementary file 1 — Figure S1 [file 41419_2021_3879_MOESM1_ESM.tif]
