# Supplementary material for: LncRNA Bmp1 promotes the healing of intestinal mucosal lesions via the miR-128-3p/PHF6/PI3K/AKT pathway
Source: Cell Death Dis. 2021 Jun 9;12(6):595. doi: 10.1038/s41419-021-03879-2 (PMC8190101; doi:10.1038/s41419-021-03879-2)
Supplement: Supplementary file 2 — Table S1 [file 41419_2021_3879_MOESM2_ESM.docx]

**Table S1. Intestinal damage scoring system^27^**

| Score | Morphologic characteristic(s) |
| --- | --- |
| 0 | No damage |
| 1 | Mild congestion, edema, smooth surface, and no ulcers |
| 2 | Hyperemia, edema, rough and granular mucosa, or intestinal adhesion |
| 3 | Severe congestion, edema, necrosis and ulcer formation on the mucosal surface, the largest ulcer diameter <1.0cm or necrotizing inflammation |
| 4 | The maximum ulcer diameter is> 1.0 cm or there is intestinal necrosis |
