# Supplementary material for: LncRNA Bmp1 promotes the healing of intestinal mucosal lesions via the miR-128-3p/PHF6/PI3K/AKT pathway
Source: Cell Death Dis. 2021 Jun 9;12(6):595. doi: 10.1038/s41419-021-03879-2 (PMC8190101; doi:10.1038/s41419-021-03879-2)
Supplement: Supplementary file 3 — Table S2 [file 41419_2021_3879_MOESM3_ESM.docx]

**Table S2. Intestinal epithelium damage scoring system^28^**

| Score | Histologic characteristic(s) |
| --- | --- |
| 1 | Only lost the tip of the villus |
| 2 | 50% villus loss |
| 3 | The entire villus disappeared, but the crypts were preserved |
| 4 | Completely lose the epithelium |
