# Supplementary material for: LncRNA Bmp1 promotes the healing of intestinal mucosal lesions via the miR-128-3p/PHF6/PI3K/AKT pathway
Source: Cell Death Dis. 2021 Jun 9;12(6):595. doi: 10.1038/s41419-021-03879-2 (PMC8190101; doi:10.1038/s41419-021-03879-2)
Supplement: Supplementary file 4 — Table S3 [file 41419_2021_3879_MOESM4_ESM.docx]

**Table S3. Sense and antisense primers for real-time reverse transcription polymerase chain reaction**

| **Genes** | **Primers** | **Sequences (5’-3’)** |
| --- | --- | --- |
| Bmp1 | Forward | GGATCCAGGGCGGCGAGAAAAGAAAG |
|  | Reverse | GTACCTTTTTTTTTTATATTGGCTGTAAC |
| PHF6 | Forward | AGAAGACAGCAGGTCCACATCCTC |
|  | Reverse | ACTTGTAATGTGCGGCTGCCTTC |
| GAPDH | Forward | TTCAACGGCACAGTCAAG |
|  | Reverse | CACCCCATTTGATGTTAGTG |
| miR-128-3p | Forward | CCGCGTCACAGTGAACCGGTCTCTTT |
| U6 | Forward | CCTGCTTCGGCAGCACA |
